# Endogenous time-varying risk aversion and asset returns

**DOI:** 10.1007/s00191-015-0435-3

**Published:** 2016-01-07

**Authors:** Michele Berardi

**Affiliations:** Economics, School of Social Sciences, The University of Manchester, Manchester, Lancashire UK

**Keywords:** Risk aversion, Returns, Asset prices, Financial markets, D83, G01, G02, G12

## Abstract

Stylized facts about statistical properties for short horizon returns in financial markets have been identified in the literature, but a satisfactory understanding for their manifestation is yet to be achieved. In this work, we show that a simple asset pricing model with representative agent is able to generate time series of returns that replicate such stylized facts if the risk aversion coefficient is allowed to change endogenously over time in response to unexpected excess returns under evolutionary forces. The same model, under constant risk aversion, would instead generate returns that are essentially Gaussian. We conclude that an endogenous time-varying risk aversion represents a very parsimonious way to make the model match real data on key statistical properties, and therefore deserves careful consideration from economists and practitioners alike.

## Introduction

The statistical analysis of price variations in financial markets has attracted a lot of attention, both from practitioners and academic economists, in an attempt to find regularities that could help us understand and possibly predict the evolution of prices in such markets. Such extensive analysis has led to the identification of a number of statistical properties for financial returns that seem to hold across markets and over time, and that can be summarized in the following set of empirical stylized facts (see, e.g., Cont 2001 and Tseng and Li 2011): i) the distribution of returns is not Gaussian but presents instead fat tails; ii) there is no serial correlation in returns; iii) there is positive correlation in absolute returns, with slow decay; iv) returns show strong volatility clustering, with large fluctuations that tend to cluster together.

Though there is agreement among researchers on such empirical observations, we still lack a clear understanding of their causes. We suggest in this work that a common origin could be identified in the time-varying nature of the risk aversion coefficient for investors, and show that an otherwise standard, rational expectations asset pricing model, once enhanced with such a feature, can generate time series for returns that replicate very closely the main stylized facts identified in the empirical literature.

A growing body of literature has recently tried to use adaptive learning to improve the empirical performance of asset pricing models. Examples include Branch and Evans (2010, 2011), Adam et al. (2015), Carceles-Poveda and Giannitsarou (2008), Bullard and Duffy (2001), Brock and Hommes (1998) and Timmermann (1993, 1996). The main success of these works has been in generating models able to show excess volatility similar to that observed in financial markets, but they have not focused on the same array of short-horizon stylized facts that we consider here.

An important attempt at explaining such short-run stylized facts comes instead from models based on stochastic interactions among traders. These models are borrowed and adapted from the physics literature, where they have been known for being able to generate data that display scaling laws regularities similar to those found in financial markets.^1^ While the spirit of these works is similar to the motivation of this paper, our study would fit perhaps better in the behavioral economics literature, as we try to remain as close as possible to a standard economics framework, while departing from it only in the way a behavioral characteristic, risk aversion, is modeled. The advantage of this approach with respect to models based on stochastic interacting particles is that the economic interpretation remains clearer. While stochastic models of interacting agents seem to be able to replicate facts such as fat tails and volatility clustering, in fact, they tend to lack microfoundations and do not explicitly provide an economic justification for the behavior of agents.

The backbone model that we use for our analysis is a simple, standard, present value asset pricing model with stochastic dividends, as presented, for example, by De Long et al. (1990). Such a model, as it stands, does a poor job in matching stylized facts about financial returns, as it implies that returns are normally distributed and independent over time. Our main contribution is to show that a simple behavioral modification of this model can generate returns that match key statistical properties of historical data for financial markets. The risk aversion coefficient for investors is usually assumed, in the standard economics and finance literature, to be a primitive of the model, a feature that is hard wired into the brain of people when they are born and that does not change. We believe instead that there is scope for modelling the attitude of agents towards risk as a feature that depends on the environment in which agents make their decisions, and that evolves with it.

For example, narrative evidence suggests that many people, who had been very cautious up to that point with their investment decisions and mainly kept their savings in government securities or similar activities, during the stock market bubble of the late nineties and early 2000 abandoned their safe investments and moved their money into more risky assets. Observing high rates of returns on stock markets, those people became more willing to take on risky activities in an attempt to join in and share the high profits that were realized on financial markets at the time. In a sort of herd-like behavior induced by their decreased risk aversion, previously cautious investors entered into the stock market. When prices then started to fall and returns decreased, those same investors became afraid of losses, their risk aversion increased and they fled financial markets, selling their assets and fuelling a sharper decrease in prices.

Alan Greenspan, on this point, said in a speech at the Federal Reserve Bank of Kansas City (Greenspan, 2005; Italics added):

“Thus, this vast increase in the market value of asset claims is in part the indirect result of *investors accepting lower compensation for risk*. [...] Any onset of *increased investor caution* elevates risk premiums and, as a consequence, lowers asset values and promotes the liquidation of the debt that supported higher asset prices.”

Greenspan suggests in his speech that changes in market values depend, partly, on changes in risk premia required by agents, which in turn depend on the attitude of investors towards risk. In this paper we make formal this argument and show that adding this feature to an otherwise standard model changes completely the statistical properties of simulated asset returns, making them similar to those observed in real markets.

Time-varying risk aversion is not new in economics. In consumption-based asset pricing models, for example, Brandt and Wang (2003) propose a time-varying risk aversion coefficient that responds to news about consumption growth and inflation, while Li (2007) studies asset prices under the assumption of a countercyclical risk aversion. We propose instead a process for risk aversion that depends on unexpected excess returns: agents adapt their attitude towards risk on the basis of the unexpected excess gains that they observe from risky activities.

While in standard economics risk aversion is a feature that depends solely on the curvature of the utility function being maximized by agents, the form of which is assumed constant over time, in behavioral economics Kahneman and Tversky (1979)’s prospect theory argues that expected utility maximization is a poor representation of how people make choices under risk, and suggests instead an alternative framework where people’s attitude towards risk is situation dependent. We will continue to use here the expected utility maximization framework, but modify it to allow for the curvature of the utility function, and therefore the risk attitude of agents, to evolve over time endogenously. An evolutionary justification for changes in attitudes towards risk is provided by Netzer (2009), who shows that, from an evolutionary perspective, the utility function of agents, and therefore their risk aversion, should depend on the probability distribution of alternatives about which agents need to make decisions. In our context, such alternatives are represented by returns from risky versus risk-free activities, and agents adapt their perceptions about the distribution of such alternatives using observations about unexpected excess returns on the stock market.

The plan of the paper is as follows: in Section 1.1 we discuss the literature related to our work, while in Section 1.2 we discuss more in detail the statistical properties of financial returns by looking at the S&P500 index as a representative case. Section 2 introduces the basic model, Section 2.1 discusses endogenous time-varying risk aversion and Section 2.2 derives the equilibrium solution for the model. Section 3 presents results from simulations of the model with constant and with endogenous time-varying risk aversion, and compares the resulting series for returns with those from real data. Section 4 discusses the results, and Section 5 concludes.

### Related literature

Two main lines of research are relevant to our work, one based on bounded rationality and learning, and the other based on behavioral models of human decisions.

In terms of the first strain of literature, Branch and Evans (2010) show that real time learning dynamics, in an otherwise standard consumption based asset pricing model, calibrated to U.S. stock data, is capable of reproducing regime-switching returns and volatilities. Branch and Evans (2011) introduce learning about risk and returns in the De Long et al. (1990) framework and show that escape dynamics emerge that look like stock market crashes, even though the escape route is not from a bubble high but from the equilibrium fundamental value. Hommes and Zhu (2014) use the concept of stochastic consistent expectations equilibrium to explain excess volatility in a standard present value asset pricing model with stochastic dividends similar to the one we consider here. Adam et al. (2015) show how adaptive learning can generate excess volatility in a consumption based asset pricing model and present an estimated version of the model to US data that can replicate some asset price puzzles such as stock price volatility, the persistence of the price-dividend ratio and the predictability of long-horizon returns. All these works mainly focus on the long-horizon properties of asset prices returns, while we will focus our attention on trying to explain and replicate statistical properties of returns in the short run.

As for the second strain of literature, Lux (2009) provides an extensive surveys on behavioral asset pricing models based on interacting agents. He divides such literature into four groups, and we follow here his classification in our discussion. A first class of models is focused on interactions between fundamentalists and chartists, where the disaggregation of markets is limited to the distinction between two classes of agents that form their demand according to different rules. Early contributions are Beja and Goldman (1980), Day and Huang (1990) and Chiarella (1992). The interaction between these two classes of agents is able to generate a mix of centripetal and centrifugal forces that can lead to rich dynamics in prices. More recently, Chiarella and He (2002, 2003) propose a model where there is heterogeneity in risk aversion between fundamentalists and trend chasers, coupled with learning about future returns, and find that the dynamics of asset prices are affected by the relative risk attitudes of different types of investors.

A second class of models is built on local stochastic interactions, based on the seminal work of Kirman (1993) on herding: here the departure from traditional economics models is more marked, with agents’ demand being determined by a local interaction mechanism by which agents are recruited by neighbors on a particular strategy. Kirman and Teyssière (2002) show that such a feature can generate long-term dependence in absolute and squared returns.

A third class of models tries to capture non-local interactions through field effects, as for example in Lux (1995, 1998): the main feature here is that traders are assumed to be influenced not by other individual agents, but by the overall mood of the market. Such models can generate fads or herding in the market that lead to autoregressive dependence in higher moments.

Finally, some authors have explored the importance of the topology of interactions on financial markets. Bouchaud and Cont (2000), for example, build on the percolation model popular in statistical mechanics and place traders on a lattice with interactions among neighboring agents forming clusters: the ensuing distribution of returns shows fat tails but higher moments are uncorrelated, thus failing to match one key stylized fact of financial markets. Subsequent works have tried to improve on this framework: for example, Iori (2002), using a different network structure, has been able to generate time series that more closely match those observed in financial markets.

Among these approaches based on stochastic interactions among agents, we believe the mechanism at work in our framework is closer in spirit to the one used in models with field effects, where individual decisions are affected by aggregate features of the economy. In our case the (individual) demand for the risky asset depends on the risk aversion coefficient, which changes endogenously in response to changes in excess returns, an aggregate feature of the market. Despite the similar interpretation, the way we model such effects, from the aggregate to the individual level and back, differs sharply, as in our case the link from macro to micro goes through an explicitly identified and endogenous response of agents’ preferences to their environment. We believe that, compared to models with field effects, our approach is more intuitive from an economic perspective.

A key feature of our model will be the time-varying nature of risk aversion. This is not new in the literature. Brandt and Wang (2003) present a model in which the coefficient of risk aversion changes in response to news about consumption growth and inflation, and find empirical support for the hypothesis that aggregate risk aversion varies in response to news about inflation. Li (2007), instead, assumes a countercyclical risk aversion that drives a pro-cyclical risk premium in asset prices, but finds that such a feature may not help explain important facts such as the predictability of long-horizon stock returns or the univariate mean-reversion of stock prices. Park (2014) proposes a model of heterogeneous risk aversion, where fundamentalists have constant risk aversion while chartists’ risk aversion varies over time due to psychological factors: the time variation in risk attitudes increases price fluctuations and generates chaotic dynamics. Finally, Smith and Whitelaw (2009) find empirical evidence in support of the hypothesis that risk aversion moves countercyclically.

An important theoretical paper for our modelling choice of risk aversion is Netzer (2009), who proposes a model of the evolution and adaptation of hedonic utility and provides an evolutionary explanation for risk attitudes that adapt according to changes in the perceived distribution of possible alternatives. We will discuss this point at length in Section 2.1.1.

In terms of evidence about short-horizon returns on financial markets, stylized facts are presented in a number of papers in the literature, such as Cont (2001) and Tseng and Li (2011). Both works show that the same stylized facts discussed in Section 1.2 below hold for a large number of financial time series, including Standard & Poor’s 500 Index, NASDAQ Composite Index and Hang Seng Index, series for individual stock prices such as IBM, Microsoft and BMW, and even series for exchange rates.

### Stylized facts

As we mentioned before, the main stylized facts identified for short-horizon returns on financial markets are: i) the distribution of returns is not Gaussian, and presents instead fat tails; ii) there is no correlation in returns; iii) there is positive correlation (with slow decay) in absolute returns; iv) returns show volatility clustering, i.e., large fluctuations tend to cluster together.

Such stylized facts hold for returns computed from many asset price series (see, e.g., Tseng and Li, 2011). As an example, we report here statistics for the S&P500 index, for the period 02/01/1957 until 12/04/2012,^2^

Returns are computed as

$$r_{t}=\frac{p_{t}-p_{t-1}}{p_{t-1}}, $$

where *p*_*t*_ is the price of the asset or index at time *t*. It is also common practice to normalize returns as follows

$$nr_{t}=\frac{r_{t}-\mu }{\sigma }, $$

where *μ* and *σ* are the mean and standard deviation of returns. Absolute returns are then defined as

$$ar_{t}=|r_{t}|. $$

**Fig. 1:**
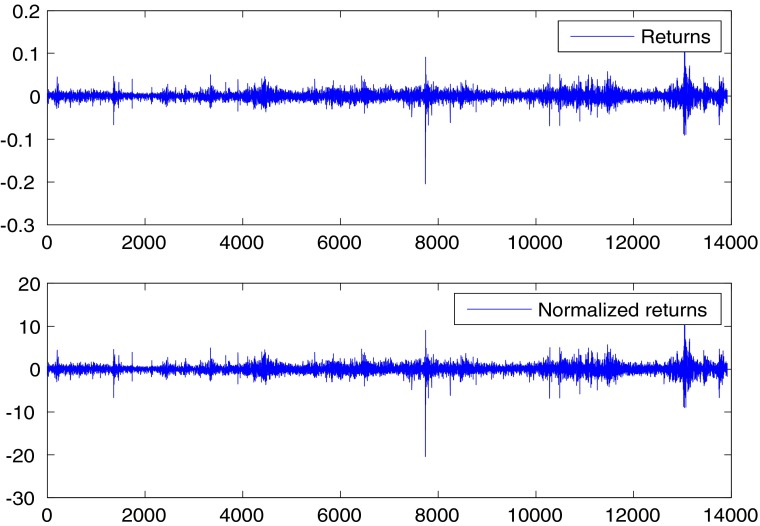
Returns and normalized returns for the S&P500 index

For the S&P500 index, *μ*_*r*_ = 0.000294 and *σ*_*r*_ = 0.0100 and returns and normalized returns are plotted in Fig. 1. It is clearly evident the volatility clustering of returns, with large movements that tend to cluster together at particular times.

**Fig. 2:**
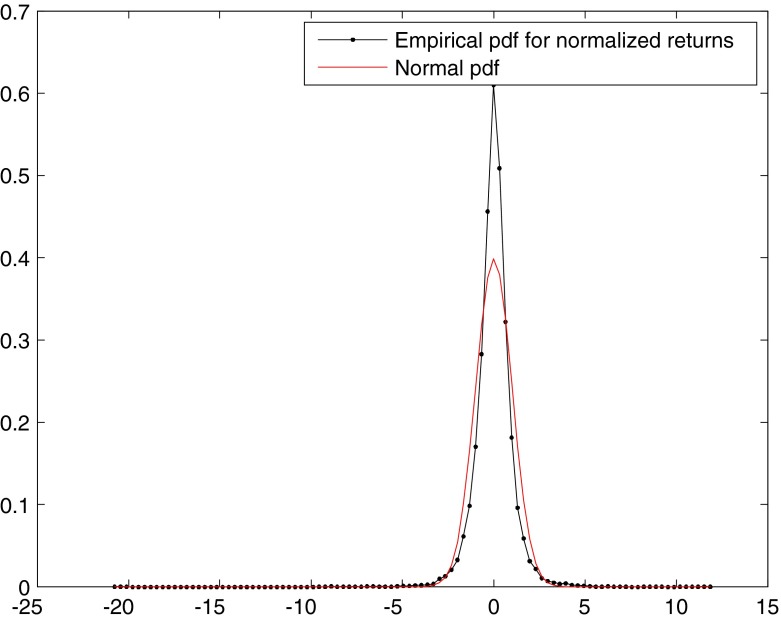
Empirical pds of normalized returns and normal fit

We compute then the empirical probability density function (pdf) for normalized returns and overlay it with the analytical normal pdf for comparison (Fig. 2): it is evident that the distribution of returns deviates from normality. Non-normality is also confirmed by the 4th moment of the distribution, kurtosis, which in the data is 24.378, while the value for a Gaussian distribution is 3, and by the Jarque-Bera test, which rejects at 5 % significance level the null hypothesis that the sample comes from a normal distribution with unknown mean and variance. Note that the estimated tail index for returns, which represents the order of the highest finite absolute moment, is 3.1083 , so the estimate for the fourth moment of the distribution is not reliable, which is consistent with previous evidence on asset returns (Cont 2001). The tail index conveys information about the tail of a distribution: the lower the index, the fatter the tails.^3^

**Fig. 3:**
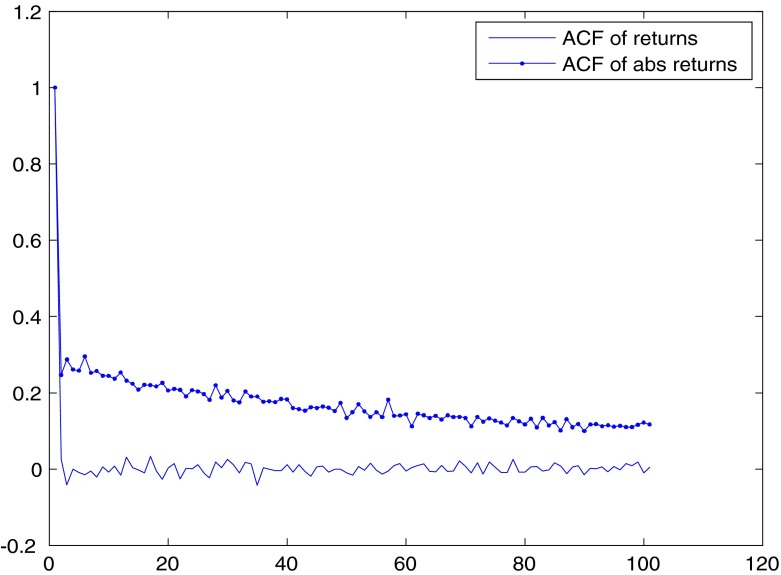
ACFs for returns and absolute returns for the S&P500 index

As for the correlation of returns and absolute returns over time, the serial correlation of returns is usually analyzed by computing the autocorrelation function (ACF). For the S&P500, the ACF for returns drops to zero after one period, while it stays positive for absolute returns at long lags, being still above .1 at 100 lags (See Fig. 3).^4^ This is a typical finding in financial markets: returns have short-memory and present exponentially decaying autocorrelations, while absolute (or squared) returns display long-memory and are characterized by hyperbolic exponential decline in autocorrelation.

We have presented in this section a brief exposition of the main stylized facts concerning statistical regularities in financial returns. We now develop a simple model that will be able to replicate closely all such facts.

## The model

We start with a standard, present value asset pricing model with stochastic dividends. There are two types of assets: a risk free one, elastically supplied, with a gross rate of return *R* = *β* ^−1^, where *β* is the discount factor; and a risky asset, the price of which is *p*_*t*_ and pays a stochastic dividend *d*_*t*_. The supply of the risky asset is exogenous and stochastic.

The dynamic equation for wealth (*W*_*t*_) is then represented by

$$W_{t+1}=RW_{t}+(p_{t+1}+d_{t+1}-Rp_{t}){z_{t}^{d}} $$

where ${z_{t}^{d}}$ is the demand for the risky asset.

Agents are myopic mean variance maximizers, and therefore maximize

1 $$ E_{t}\left[ \alpha_{t}E_{t}W_{t+1}-{\alpha_{t}^{2}}/2Var_{t}(W_{t+1})\right] $$

where *E*_*t*_ and *V* *a* *r*_*t*_ are the conditional expectation and variance of wealth based on the subjective probability distribution of agents, and *α*_*t*_∈*R* is the coefficient of absolute risk aversion.

The coefficient of risk aversion has a time *t* subscript, to make it explicit that we will allow such parameter to evolve over time: the specification of its endogenous dynamics will be given in the next section. Though risk aversion evolves over time, at each time *t* agents take such a coefficient as given in their maximization problem, only to revise it in the following period on the basis of new evidence. Moreover, by assuming such a parameter as fixed, agents also implicitly disregard the feedback from time-varying risk aversion on asset price volatility, and maintain this parameter constant in their maximization problem. In this respect, agents in our setting implement an anticipated utility model in the spirit of Kreps (1998). Such a modeling strategy has been largely adopted in the macroeconomics literature on bounded rationality and learning (e.g., Sargent 1993, 1999 and Evans and Honkopohja 2001) and we believe it can represent a good approach also in the present context where behavioral parameters rather than beliefs evolve over time.

Note also that risk aversion is allowed to become negative, thus making agents risk lovers: this will happen in our setting when there is a long series of large and unexpected profits in the stock market, so that investors become, in effect, willing to gamble in financial markets. 5

From the above setting, it follows that the optimal demand for the risky asset is given by

2 $$ {z_{t}^{d}}=\frac{E_{t}\left( p_{t+1}+d_{t+1}\right) -\beta^{-1}p_{t}}{\alpha_{t}{\sigma_{t}^{2}}}, $$

where ${\sigma _{t}^{2}}$ is agents’ conditional variance of excess returns *p*_*t*+1_ + *d*_*t*+1_−*R* *p*_*t*_ and is given by

3 $$ {\sigma_{t}^{2}}=E_{t}\left[ \left( p_{t+1}+d_{t+1}\right) -E_{t}\left( p_{t+1}+d_{t+1}\right) \right]^{2}. $$

Equating demand and supply, denoted by ${z_{t}^{s}}$, we obtain the pricing equation

4 $$ p_{t}=\beta E_{t}\left( p_{t+1}+d_{t+1}\right) -\beta \alpha_{t}\sigma_{t}^{2}{z_{t}^{s}}. $$

The exogenous process for dividends is assumed to be given by

5 $$ d_{t}=d_{0}+u_{t}, $$

where *d*_0_ is a constant and *u*_*t*_ is an i.i.d., zero mean, normally distributed disturbance.

We also assume that supply follows the exogenous random process

6 $$ {z_{t}^{s}}=s_{0}+v_{t}, $$

where *s*_0_ is a constant and *v*_*t*_ is an i.i.d., zero mean, normally distributed disturbance uncorrelated with *u*_*t*_.

### Endogenous time-varying risk aversion (ETVRA)

The coefficient of absolute risk aversion, *α*_*t*_, is modelled as time-varying and endogenous, depending on unexpected (excess) returns in the stock market: higher returns than expected make agents more willing to take on the risk involved in investing in the stock market. Specifically, we postulate a process of the form

7 $$ \alpha_{t}=\alpha_{t-1}-\gamma_{\alpha }\pi_{t}, $$

where *γ*_*α*_≥0 and

8 $$ \pi_{t}=p_{t}+d_{t}-E_{t-1}(p_{t}+d_{t}), $$

i.e., risk aversion decreases when excess profits in the financial market are higher than expected.^6^ Parameter *γ*_*α*_ represents the sensitivity of risk aversion to unexpected returns: with *γ*_*α*_ = 0, we have the standard case of constant risk aversion over time, while positive values of *γ*_*α*_ mean that agents are willing to take on more risk when they see excess returns in the stock market compared to what they expected (and vice-versa). Such endogenous dynamics for the coefficient of risk aversion will make the reduced form parameters in the solution for prices be time-varying.

#### An evolutionary justification to ETVRA

The postulated process for risk aversion dynamics, represented by Eq. 7, might seem ad-hoc at first. In reality, it has a simple and intuitive justification under an evolutionary framework for the derivation of the utility function of agents coupled with learning dynamics.

From an evolutionary perspective, it can be shown (Netzer 2009) that, if individual decisions are guided by hedonic utility, i.e., if agents, given a set of alternatives, prefer the one that promises the greatest pleasure, then a utility function can be considered as a reward system to induce agents to make optimal choices, and its slope can be interpreted as the degree of attention devoted to the available alternatives. The shape of the utility function therefore determines where agents put more attention in their choices. Evolutionary forces would shape the utility function to make it responsive to the decision environment and, in particular, to the distribution of alternatives available for choice, in order to devote more attention to areas where the decision is more critical. If the properties of such distribution are not known a-priori, agents would have to learn them from the environment, and the evolution of agents’ beliefs about such distribution would then affect the shape of their utility function, making it time-varying in a way that is well represented by our ETVRA dynamics.

In our setting, the distribution of excess returns from the risky activity is unknown, and agents adapt their beliefs about it over time by moving in the direction indicated by unexpected returns. Equation 7 can, therefore, be interpreted as a simple adaptive learning rule with constant gain, through which agents keep track of the evolution of returns on the stock market by adjusting their beliefs in the direction suggested by forecast errors. A shift in probability is signalled by *π*_*t*_, a realization of unexpected excess returns on the stock market. Parameter *γ*_*α*_ represents a behavioral feature that captures the degree of adjustment of agents’ perceived distribution of excess returns to evidence about unexpected realizations. As beliefs about the distribution of returns adapt, so does risk aversion. In particular, a decrease in the estimate of *α*, which corresponds to a shift in probability mass to alternatives with larger payoffs, reduces risk aversion.

### Equilibria

In this section, we solve the model for an equilibrium under constant risk aversion, and then introduce time-varying risk aversion dynamics. Under rational expectations (RE) and constant risk aversion, two possible equilibria exist: a fundamental equilibrium, where pice dynamics are affected only by the fundamental features of the economy, and a bubble solution, which admits sunspot components. For a number of reasons discussed below, we will focus only on the fundamental equilibrium.

#### Constant risk aversion

Under RE, with constant risk aversion (*α*_*t*_≡*α*), it is well known that Eqs. 4–6 admit two possible equilibria, the fundamental solution and the bubble one.

The price equation in the fundamental equilibrium is represented by

9 $$ p_{t}=\beta \left( 1-\beta \right)^{-1}\left( d_{0}-\alpha s_{0}\sigma^{2}\right) -\beta \alpha \sigma^{2}v_{t}, $$

while, in the bubble equilibrium, it is given by

10 $$ p_{t}=\alpha \sigma^{2}s_{0}-d_{0}+\beta^{-1}p_{t-1}-\alpha \sigma^{2}v_{t-1}+\xi_{t}, $$

where *ξ*_*t*_ is a martingale difference sequence that opens the door to sunspot variables affecting prices. It is also known (see, e.g., Branch and Evans, 2011) that the fundamental equilibrium is adaptively learnable by agents, while the bubble one is not. Moreover, since *β*<1, the process for *p*_*t*_ in the bubble equilibrium is not stable.

#### ETVRA

With a time-varying risk aversion, the solution to the model must be enriched to include an equation for the evolution of risk aversion and for the variance of returns. We stress again here that agents in our setting, in the spirit of the anticipated utility model of Kreps (1998), solve at each point in time their maximization problem (and we, as modeler, find the equilibrium) as if risk aversion and the conditional variance of dividends were constant, but then modify their attitudes towards and perception of risk as new evidence on unexpected excess returns comes available next period.

For the fundamental equilibrium, the evolution of the system is therefore represented by

11 $$\begin{array}{@{}rcl@{}} p_{t} &=&\beta \left( 1-\beta \right)^{-1}\left( d_{0}-\alpha_{t}s_{0}{\sigma_{t}^{2}}\right) -\beta \alpha_{t}{\sigma_{t}^{2}}v_{t} \end{array} $$

12 $$\begin{array}{@{}rcl@{}} \alpha_{t+1} &=&\alpha_{t}-\gamma_{\alpha }\pi_{t}, \end{array} $$

13 $$\begin{array}{@{}rcl@{}} \pi_{t} &=&-\beta \alpha_{t}{\sigma_{t}^{2}}v_{t}+u_{t} \end{array} $$

14 $$\begin{array}{@{}rcl@{}} {\sigma_{t}^{2}} &=&\frac{1\pm \sqrt{1-4{\alpha_{t}^{2}}\beta^{2}\sigma_{v}^{2}{\sigma_{u}^{2}}}}{2{\alpha_{t}^{2}}\beta^{2}{\sigma_{v}^{2}}} \end{array} $$

where Eq. 13 for unexpected excess returns comes from computing (8) in the fundamental equilibrium. Expression (14) for the conditional variance of excess returns is obtained from Eq. 3 by substituting in the equations for prices and dividends.

By substituting into expression (12) for *α*_*t*+1_ those for *π*_*t*_ and ${\sigma _{t}^{2}}$, it is possible to see that the dynamics for the risk aversion coefficient are represented by a non linear difference equation that depends on parameters *β* and *γ*_*α*_ and on the variances and realizations of the two processes *u*_*t*_ and *v*_*t*_:

$$\alpha_{t+1}=\alpha_{t}-\gamma_{\alpha }\left[ u_{t}-\beta \alpha_{t} \frac{1\pm \sqrt{1-4{\alpha_{t}^{2}}\beta^{2}{\sigma_{v}^{2}}{\sigma_{u}^{2}}} }{2{\alpha_{t}^{2}}\beta^{2}{\sigma_{v}^{2}}}v_{t}\right] . $$

Simulations will be done with the (-) root, as this is the one that Branch and Evans (2011) show to be stable under learning. It is also the one that ensures that simulated prices follow a smooth process without unrealistic and sudden large jumps.

In the bubble equilibrium, instead, the evolution of the system under ETVRA is described by

$$\begin{array}{@{}rcl@{}} p_{t} &=&\left( \alpha_{t-1}\sigma_{t-1}^{2}s_{0}-d_{0}\right) +\beta^{-1}p_{t-1}-\alpha_{t-1}\sigma_{t-1}^{2}v_{t-1}+\xi_{t}, \\ \alpha_{t+1} &=&\alpha_{t}-\gamma_{\alpha }\pi_{t}, \\ \pi_{t} &=&u_{t} \\ {\sigma_{t}^{2}} &=&{\sigma_{u}^{2}}+\sigma_{\xi }^{2}. \end{array} $$

It is easy to see that, in this case, *α*_*t*_ simply follows a random walk process

$$\alpha_{t+1}=\alpha_{t}-\gamma_{\alpha }u_{t}. $$

In a bubble equilibrium, therefore, asset prices diverge, unexpected excess returns from the risky activity are white noise and the ETVRA follows a random walk: for all these reasons, we do not focus on such equilibrium in this work. In a fundamental equilibrium, instead, asset prices follow a stationary process, but unexpected excess returns depend on risk aversion of agents and the dynamics for the ETVRA coefficient are highly nonlinear. These features, we will show, impact significantly on the implied nature of returns from the risky asset, and are able to generate dynamics that in many dimensions closely resemble those observed in real financial markets.

## Simulations and statistical analysis

In order to compare the statistical properties of observed returns on financial markets with those that come from our model, we simulate price dynamics in the fundamental RE equilibrium, first with constant risk aversion and then with ETVRA. From such series, we compute returns and analyze their properties in comparison with the stylized facts reported in Section 1.2.

The baseline calibration for the model is as follows: *β* = .95, *s*_0_ = 1 , *d*_0_ = 5 , ${\sigma _{v}^{2}}=.5$, ${\sigma _{u}^{2}}=.9$. These parameter values are taken from Branch and Evans (2011), apart from *d*_0_ which we have set to a higher value in order to avoid prices hitting the zero lower bound.^7^ We also set the initial value for risk aversion, *α*_0_, equal to 0.75, which is the value used by Branch and Evans (2011) for their constant risk aversion. Sensitivity analysis results regarding these parameters are reported in Section 3.3. The key new parameter *γ*_*α*_ is instead calibrated to 0.025: extensive investigations have shown that the higher is *γ*_*α*_, the further away from normality is the distribution of returns. We will discuss further this point later on when reporting results from simulations.

### Asset returns with constant risk aversion

Under constant risk aversion, it is clear from Eq. 9 that returns follow a normal distribution. For comparison with subsequent statistics under ETVRA, we simulate our model with constant risk aversion and compute returns from the resulting time series for asset prices. Mean and standard deviation of simulated returns, $\tilde {\mu }_{t}$ and $\tilde {\sigma }_{t}$, are, respectively, 0.000119 and 0.0156, in line with those found in real data.

Normality of simulated returns is confirmed by the Jarque-Bera test, which can not reject at 5 % significance level the null hypothesis that the sample comes from a normal distribution with unknown mean and variance, and by the estimated kurtosis, which is 3.0049. Moreover, computing the ACFs for simulated returns and their absolute value we find that, in both series, the autocorrelation function drops to zero quickly, in contrast to what real returns show.

All the above evidence on simulated returns under constant risk aversion clearly indicates that the model with constant risk aversion is not able to replicate the key stylized facts that have been consistently observed in series for returns on real financial markets.

### Asset returns with ETVRA

We simulate now the model with endogenous time-varying risk aversion and compute returns from the resulting time series for asset prices. Mean and standard deviation of simulated returns, $\tilde {\mu }_{t}$ and $\tilde {\sigma }_{t}$, are, respectively, 0.000090 and 0.0110, in line with those found in real data.

**Fig. 4:**
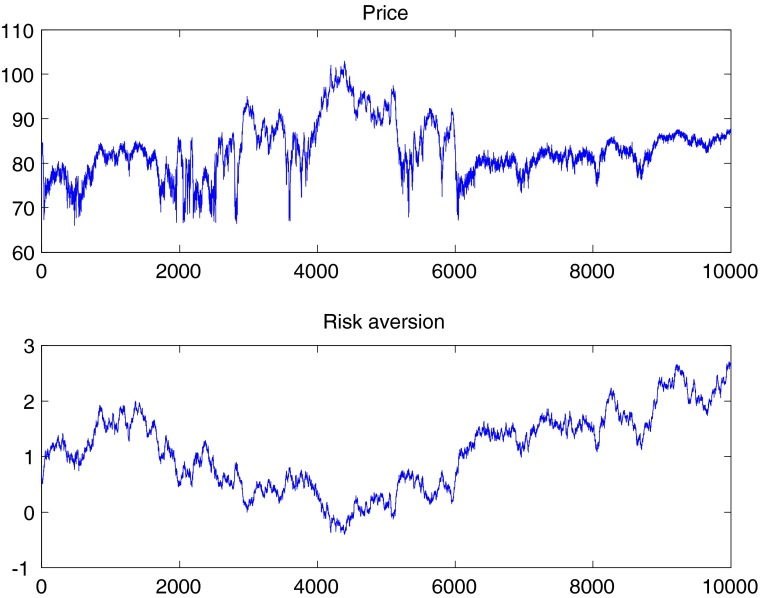
Evolution of prices and risk aversion under ETVRA. Parameter values and initial conditions as follows: *β* = .95, *s*_0_ = 1, *d*_0_ = 5, $\sigma _{v}^{2}=.5$, ${\sigma _{u}^{2}}=.9$, *α*_0_ = 0.75

We first show in Fig. 4 the time series of prices and risk aversion coefficient under time-varying risk aversion for a representative run of simulated data. It is possible to see that ETVRA introduces persistence in prices, which with constant risk aversion would simply be white noise around the fundamental equilibrium price - see Eq. 9.

**Fig. 5:**
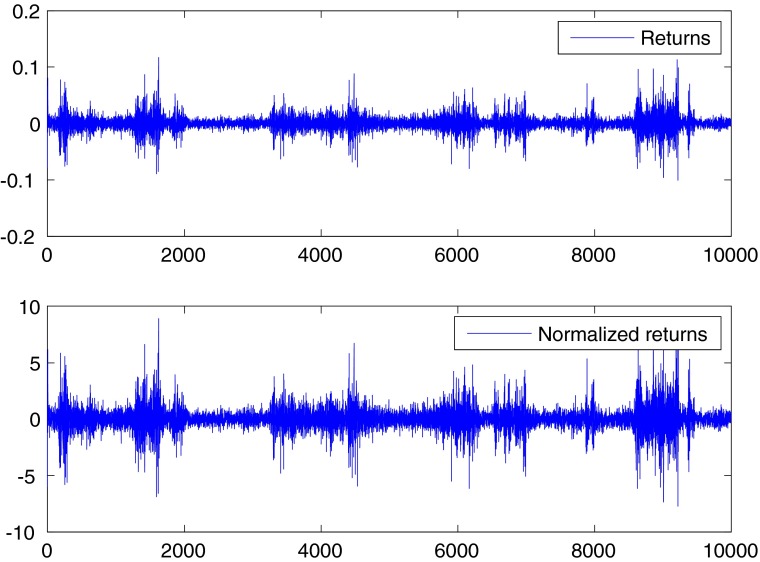
Returns and normalized returns for the simulated series with ETVRA. Parameter values and initial conditions as follows: *β* = .95, *s*_0_ = 1, *d*_0_ = 5, $\sigma _{v}^{2}=.5$, ${\sigma _{u}^{2}}=.9$, *α*_0_ = 0.75

We then plot returns from the same representative run of simulated data in Fig. 5, together with their normalized counterpart. It is evident now that ETVRA generates volatility clustering in returns.

**Fig. 6:**
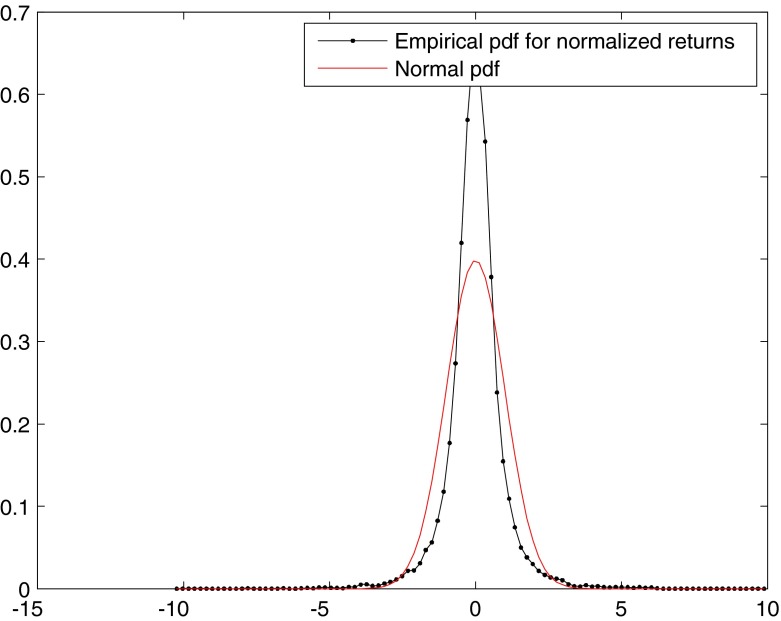
Empirical pds of simulated normalized returns and normal fit

In Fig. 6, we compute the empirical pdf for the simulated normalized returns and overlay it with the analytical normal pdf for comparison: it seems evident that the distribution of returns under ETVRA differs significantly from a Gaussian and shows instead characteristics similar to those observed for returns in real financial markets. This impression is confirmed by the Jarque-Bera test, which this time rejects at 5 % significance level the null hypothesis that the sample comes from a normal distribution with unknown mean and variance. Also, in terms of kurtosis, we find a value of 21.48, in line with the one estimated for S&P500 returns and much higher than the one, close to 3, obtained under constant risk aversion and consistent with a Gaussian distribution. Note, though, that the estimated tail index is 2.88, so the estimated fourth moment of the distribution is not reliable.

**Fig. 7:**
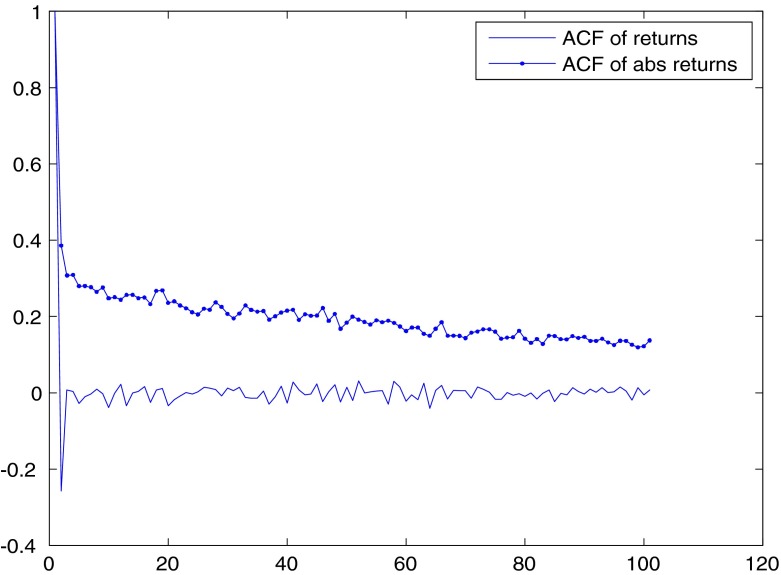
ACFs for returns and absolute returns for the simulated series with ETVRA

We note here that estimated kurtosis for the distribution of simulated returns is sensitive to the value of *γ*_*α*_: higher values of *γ*_*α*_ imply higher kurtosis, as the distribution of returns drifts further away from normality.^8^ As risk aversion becomes more sensitive to unexpected excess returns from the risky asset, the feedback effect from returns to asset demand and therefore prices is strengthened, making the distribution of returns more heavy tailed. Figure 7 plots the ACFs for simulated returns under ETVRA and for their absolute value: we can see that, while there is no serial correlation in returns, absolute returns show positive correlation with slow decay, very similar to that observed in S&P500 returns. All the above evidence on simulated returns with ETVRA shows that simply introducing an endogenous risk aversion coefficient that responds to unexpected excess returns from the risky activity in an otherwise standard asset pricing model fundamentally changes the statistical properties of simulated returns, and makes them strikingly resemble those observed in real financial markets.

### Robustness check

In order to investigate the sensitivity of our results to the specific values assumed for the parameters in the model, we run now extensive simulations under different parameterization: reported statistics are computed as averages over 100 simulations, each of 10,000 time periods.

**Table:** 

Model	Mean	Std. Dev.	Kurtosis	Tail index	ACFs
SimpleParameterization					(1/5 lags)
Baseline*	8.5910 × 10^−5^	0.0115	23.1789	2.8815	0.4166/0.3346
*γ*_*α*_ = .005	8.1076 × 10^−5^	0.0113	7.1184	4.6997	0.3778/0.2300
*γ*_*α*_ = .1	1.5112 × 10^−4^	0.0146	74.8951	2.2048	0.5184/0.4329
*s*_0_ = 5	9.7316 × 10^−5^	0.0118	21.5593	2.8856	0.4132/0.3191
*d*_0_ = 1	−3.48 × 10^−2^	20.5120	5.2203 × 10^3^	1.1202	0.0388/0.0423
${\sigma _{u}^{2}}=.1$	6.1165 × 10^−7^	0.0011	5.1893	5.4159	0.3430/0.1786
${\sigma _{u}^{2}}=.5$	3.5291 × 10^−5^	0.0075	12.1375	3.5280	0.3972/0.2945
${\sigma _{v}^{2}}=.1$	2.4291 × 10^−4^	0.0200	54.9828	2.6201	0.4039/0.3240
${\sigma _{v}^{2}}=.9,{\sigma _{u}^{2}}=.1$ **	1.7943 × 10^−6^	0.0016	5.5993	5.0826	0.3591/0.1931

*Baseline parameterization: *β* = .95, *s*_0_ = 1, *d*_0_ = 5, *γ*_*α*_ = .025, ${\sigma _{u}^{2}}=.9$, $\sigma _{v}^{2}=.5 $. Each raw differs from baseline parameterization only in the element indicated

** ${\sigma _{u}^{2}}$ had to be adjusted in order to avoid ${\sigma _{t}^{2}}$ from Eq. 14 to become a complex number

The first thing to notice is that results for the baseline parameterization reported in the previous section are confirmed when averaged across 100 simulations: mean returns are effectively zero, with a variance around .01 and kurtosis close to the value found in empirical data.

In terms of the effect of different parameter values on the properties of simulated returns, a few comments are necessary. First, in terms of the variances of the stochastic terms in the model, the variance of dividends (${\sigma _{u}^{2}}$) needs to be high enough to generate kurtosis and tail indexes in line with those observed in the data: low values of $\sigma _{u}^{2}$ in fact generate thinner tails. The variance of risky asset supply (${\sigma _{v}^{2}}$) also needs to be sufficiently high but not too high: in this case, though, low values of ${\sigma _{v}^{2}}$ generate the opposite problem, i.e., fatter tails (but note that the product of the two variances must be such that ${\sigma _{t}^{2}}$ remains real). The different impact of the two variances can be understood by looking at Eq. 14 for the conditional variance of dividends. While $\sigma _{u}^{2}$ enters at the numerator with a multiplicative effect on the time varying risk aversion, the main effect of ${\sigma _{v}^{2}}$ comes from the term it the denominator (again with a multiplicative effect on *α*_*t*_ ): while higher values for the first thus enhance the impact of unexpected returns (see also Eq. 13) on risk aversion and thus demand, higher values for the second dampen the effect.

Second, in terms of the constants in the model, *s*_0_ (average supply) is fairly irrelevant, while *d*_0_ (average dividends) needs to remain high enough (roughly above 2) to avoid negative prices and ensuing unrealistic statistics.

An important thing to note, also, is that all these parameterizations imply distributions of returns that pass the JB Test for non-normality (i.e., the null of normality is rejected at 5 % significance level). Estimated tail indexes are also in general between two and five, consistent with evidence on asset returns.^10^ ACFs of absolute returns also remain positive and significant at long lags, and tend to be higher with parameterizations that generate fatter tails.

We have already discussed the effect of *γ*_*α*_, but it is worth stressing here the point once again. This parameter represents the degree of adjustment of risk aversion to unexpected excess returns. When equal to zero, we have the standard model with constant risk aversion and simulated results are as reported in Section 3.1; as *γ*_*α*_ increases, the endogenous character of risk aversion is strengthened and the tails of the return distribution gets fatter, which is reflected in a higher kurtosis and smaller tail index. Interpreting Eq. 7 as a simple adaptive learning rule, this means that *γ*_*α*_ represents a constant gain coefficient in the learning algorithm. Milani (2011), in a model of business cycles where agents need to learn about inflation, output and interest rates, has estimated a gain coefficient of about 0.02.^11^ A similar value was estimated also in Milani (2007). Though in these works the learning algorithm is more complex than the one we use here, as it is basically a recursive least squares algorithm with constant gain, similar values for the gain in our setting seem to generate returns with characteristics that best resemble those found in real data.

## Discussion

Comparing simulations of returns with a constant and a time varying risk aversion, the differences in the statistical properties of the generated time series are remarkable. With a constant risk aversion, returns are essentially normally distributed, and fail to show any of the properties identified as stylized facts for returns on financial markets. In contrast, with ETVRA, the generated returns match surprisingly well the statistical properties of actual returns.

Our simulations thus suggest that ETVRA could be an important factor in understanding financial markets. An endogenous risk aversion coefficient that responds to excess returns introduces a reinforcement effect from market returns to asset demand and prices, and can, therefore, generate fat tail distributions for returns. We have also shown that as such a reinforcement effect is strengthened (i.e., as *γ*_*α*_ is increased) and the impact of excess returns on asset demand increased, the tails of the distribution get fatter.

In light of the literature discussed in the Introduction, we believe the mechanism at work in our setting could be considered a sort of field effect, in the sense that it introduces a feedback loop from the aggregate to individual decisions. In models with field effects, individual behavior is affected by some aggregate feature of the economy, giving rise to herds: in a similar way here, unexpected excess returns, by decreasing risk aversion, impact individual demand and result in a herd-like behavior: when asset prices rise more than expected, demand increases and, as a consequence, prices are increased even further, reinforcing the feedback effect and generating heavy tails in the distribution of returns.

Such a feedback effect from unexpected returns to demand can explain the correlation in absolute returns paired with an absence of correlation in returns. Absolute returns are, in fact, a measure of volatility, so the fact that they are correlated while returns are not means that there is predictability in the volatility of returns but not in returns themselves. The endogenous time-varying risk aversion in our model does not affect expected returns (which are still zero), but generates volatility clustering as periods of high volatility (low risk aversion) and of low volatility (high risk aversion) alternate. Low risk aversion generates higher volatility because it amplifies the impact of prices on demand, as can be seen in Eq. 2. This mechanism is thus able to generate fat tails in the distribution of returns and correlation in their volatility by endogenizing the demand through a link to the unexpected component of returns.

From a technical point of view, time-variation of risk aversion transforms the baseline additive stochastic framework into a doubly stochastic, multiplicative process that is able to generate fat tailed distributions. 12

## Conclusions

We have proposed in this paper a possible unifying explanation for many stylized facts about returns on financial markets that had so far eluded a common understanding. Our suggestion is that features such as volatility clustering, non-normality of distribution and heavy tails, absence of correlation in returns but positive correlation (with slow decay) in their absolute value, can all emerge in an otherwise standard, present value model of asset prices with stochastic dividends if an endogenous time-varying risk aversion is introduced. Simulations of the model with a coefficient of risk aversion that is allowed to change endogenously in response to unexpected excess returns in the risky activity produced returns that are surprisingly close, in all these dimensions, to real data, while simulated returns from the same model under constant risk aversion display normality of distribution, absence of volatility clustering and lack of correlation both in returns and in their absolute value. We conclude that endogenous time-varying risk aversion represents a very parsimonious way to explain key stylized facts in financial markets and deserves careful consideration from economists and practitioners alike.

## Footnotes

1 An extensive survey of these models is provided in Lux (2009).

2 Data are freely available at: http://research.stlouisfed.org/fred2/series/SP500?cid=32255

3 The tail index is computed using the Hill estimator (Hill 1975), based upon the upper 2.5 % tail of the half sample. Estimates of the tail index are dependent on the sample fraction used: we choose to report here the statistics based on the half sample after experimenting with different fractions, always getting values between 2 and 4.

4 Lux (2009) reports that, in fact, the ACF for the S&P500 absolute returns has been found to remain positive over 2500 lags.

5 Note that such a feature is not essential for our results. We tried a different specification where risk aversion is constrained to remain positive and all the results of the paper carried through.

6 Note that *E*_*t*−1_ *β* *p*_*t*−1_ = *β* *p*_*t*−1_, so these terms drop out of Eq. 8.

7 Branch and Evans (2011) avoid such a problem by modelling supply slightly differently from us and allowing it to become endogenous when prices sharply decline: this is meant to capture the drying up of asset float in financial markets that perform poorly. We chose not to use such a mechanism here, but simulations showed that it would not affect our results.

8 The case with constant risk aversion corresponds to *γ*_*α*_ = 0, and we have seen in Section 3.1 that it implies normality of returns and a kurtosis close to 3.

10 Again, the main exception being for *d*_0_ = 1, which, as noted before, generates unrealistic statistics.

11 More precisely, he estimated a value of 0.0196, with a 95 % credible interval between 0.015 and 0.025.

12 The general formal framework for such processes was first studied by Kesten (1973).
